# Relationships among Common Illness Symptoms and the Protective Effect of Breastfeeding in Early Childhood in MAL-ED: An Eight-Country Cohort Study

**DOI:** 10.4269/ajtmh.17-0457

**Published:** 2018-01-29

**Authors:** Stephanie A. Richard, Benjamin J. J. McCormick, Jessica C. Seidman, Zeba Rasmussen, Margaret N. Kosek, Elizabeth T. Rogawski, William Petri, Anuradha Bose, Estomih Mduma, Bruna L. L. Maciel, Ram Krishna Chandyo, Zulfiqar Bhutta, Ali Turab, Pascal Bessong, Mustafa Mahfuz, Laura E. Caulfield, on behalf of the MAL-ED Network Investigators

**Affiliations:** 1 Fogarty International Center/National Institutes of Health, Bethesda, Maryland;; 2 Johns Hopkins University, Baltimore, Maryland;; 3 University of Virginia, Charlottesville, Virginia;; 4 Christian Medical College, Vellore, India;; 5 Haydom Lutheran Hospital, Haydom, Tanzania;; 6 Federal University of Ceara, Fortaleza, Brazil;; 7 Tribhuvan University, Kathmandu, Nepal;; 8 Aga Khan University, Karachi, Pakistan;; 9 University of Venda, Thohoyandou, South Africa;; 10 icddr,b, Dhaka, Bangladesh

## Abstract

Children in low-income countries experience multiple illness symptoms in early childhood. Breastfeeding is protective against diarrhea and respiratory infections, and these illnesses are thought to be risk factors of one another, but these relationships have not been explored simultaneously. In the eight-site MAL-ED study, 1,731 infants were enrolled near birth and followed for 2 years. We collected symptoms and diet information through twice-weekly household visits. Poisson regression was used to determine if recent illness history was associated with incidence of diarrhea or acute lower respiratory infections (ALRI), accounting for exclusive breastfeeding. Recent diarrhea was associated with higher risk of incident diarrhea after the first 6 months of life (relative risk [RR] 1.10, 95% confidence interval [CI] 1.04, 1.16) and with higher risk of incident ALRI in the 3- to 5-month period (RR 1.23, 95% CI 1.03, 1.47). Fever was a consistent risk factor for both diarrhea and ALRI. Exclusive breastfeeding 0–6 months was protective against diarrhea (0–2 months: RR 0.39, 95% CI 0.32, 0.49; 3–5 months: RR 0.83, 95% CI 0.75, 0.93) and ALRI (3–5 months: RR 0.81, 95% CI 0.68, 0.98). Children with recent illness who were exclusively breastfed were half as likely as those not exclusively breastfed to experience diarrhea in the first 3 months of life. Recent illness was associated with greater risk of new illness, causing illnesses to cluster within children, indicating that specific illness-prevention programs may have benefits for preventing other childhood illnesses. The results also underscore the importance of exclusive breastfeeding in the first 6 months of life for disease prevention.

## INTRODUCTION

Children in low-income countries are exposed to numerous infections in early life either concurrently or within a short timeframe because of high rates of infectious diseases and pathogens in their environments, poor nutritional status, and immune function, and lack of access to health care. Studies of childhood illness often focus on a specific disease in a population,^1^ and mortality studies often attribute a single cause of death, even if multiple illnesses were present.^2^ Comorbidities in low-income populations have not been fully explored, nor has the distribution of illnesses over time.

Illnesses are not likely to be evenly distributed in a population. Prevalence and incidence estimates can be misleading as they represent an average burden of disease in a population; however, many children may not experience the illness at all, whereas other children may have persistent or recurring illnesses. In addition, children may experience multiple illnesses concurrently or consecutively, increasing the risk of severe disease or death.^3^ If there are particularly vulnerable children within a population who experience multiple illnesses or a greater number of days with illnesses, targeting interventions to them may more efficiently improve the health of the community.

Lower respiratory diseases and diarrhea are common ailments in children in low-income countries, and, after neonatal causes, continue to be the second and third leading causes of mortality in children under the age of five.^2^ Importantly, there is evidence that diarrhea is a risk factor for lower respiratory infections,^4–6^ highlighting the risks of consecutive or comorbidities as the prevalence of any single illness fails to give a complete picture of the infectious disease burdens. Exclusive breastfeeding has been found to protect against both diarrhea^7–13^ and acute lower respiratory infections (ALRI),^9,14^ but little has been published that considers both the history of illness and breastfeeding practices, and how those factors relate to risk of ALRI and diarrhea.

The Etiology, Risk Factors, and Interactions of Enteric Infections and Malnutrition and the Consequences for Child Health and Development (MAL-ED) study is a longitudinal cohort in eight study sites.^15^ MAL-ED field sites collected caregiver reports of the daily presence of symptoms the child experienced from near birth to 24 months of age.^16^ Together, these data enable us to identify the most common symptoms co-circulating in these communities, relate illnesses defined within the study to those observed and reported by caregivers, and determine temporal relationships and coincidence of different symptoms over time. In addition, we are able to determine if the illness burden in a site is driven by a subset of vulnerable children and estimate the relationship between recent history of diarrhea, ALRI, or fever and future risk of episodes.

## METHODS

The MAL-ED study was conducted at eight different sites from November 2009 to February 2014: Bangladesh (Dhaka: BGD), India (Vellore: INV), Nepal (Bhaktapur: NEB), Pakistan (Naushehro Feroze: PKN), Brazil (Fortaleza: BRF), Peru (Loreto: PEL), South Africa (Venda: SAV), and Tanzania (Haydom: TZH).^15^ Children were enrolled within 17 days of birth and visited twice a week by well-trained fieldworkers who collected daily information about symptoms using harmonized and standardized data collection forms.^16^ Field workers asked caregivers if the child was ill or had any symptoms for each day since the last visit. On average, caregivers were visited in their homes 99 times per year (means at the sites ranged from 92 to 101 visits per year) to inquire about the last 3 days. Symptom prevalence per child was calculated as the sum of days with a symptom divided by the days followed in the study, multiplied by 100.

The MAL-ED protocol defined diarrhea as three or more loose stools in 24 hours, or at least one loose stool with blood.^17^ Diarrhea episodes were separated by two diarrhea-free days.^18^ The study definition of ALRI was met when a child had 1) cough or shortness of breath (on the day of the visit or the previous day) and 2) a rapid respiratory rate on the day of the visit (average of two measurements taken by the field worker).^19^ A rapid respiratory rate was defined according to the age of the child using World Health Organization (WHO) guidelines (< 60 days old: ≥ 60 breaths/minute; 60–364 days: ≥ 50 breaths/minute; and ≥ 365 days of age: ≥ 40 breaths/minute).^20^ ALRI episodes were separated by 14 ALRI-free days. The daily number of symptoms was calculated by adding together symptoms reported on each day. Fieldworkers also recorded instances of hospitalization.

Breastfeeding and other basic feeding characteristics were recorded for the day before the fieldworker visit.^21^ In this cohort, < 60% of children were exclusively breastfed (EBF) beyond 1 month of age,^22^ although often mothers reported giving non-breast milk foods to their children and then returning to exclusive breastfeeding.^23^ Because of this, to evaluate the role of exclusive breastfeeding on disease risk, for each day of follow-up, we calculated the percentage of days with exclusive breastfeeding during the past 30 days and then categorized feeding based on whether they were exclusively breastfed less than, or more than or equal to 50% of the previous month.

Weight was measured at baseline and at each month of age. Weight-for-age *z*-scores (WAZ) were calculated using the WHO program for Stata version 12.1 (StataCorp, College Station, TX).

The MAL-ED project generated a socioeconomic status (SES) composite indicator variable that could be compared across study sites. The SES construct combines access to improved Water and sanitation, Assets, Maternal education, and average monthly household Income into a score (WAMI) that ranges between 0 and 1.^24^ The SES variables were collected at 6, 12, and 18 months, and given the low temporal variability, the WAMI values were averaged for each child. We also collected information on the mother’s age and the number of children the mother had at the time of her infant’s enrollment into the cohort.

To determine if the symptoms were distributed evenly throughout the cohorts or if they clustered in vulnerable children, we produced cumulative distribution plots to visually compare the percent of children in each site with the percent of days with illness that each child contributed. Separate plots were produced for any major symptom (any day with ALRI, diarrhea, cough, fever, or vomiting), diarrhea, and ALRI. Because more common symptoms are found more frequently in combination with other symptoms by chance alone, we assessed the likelihood of comorbidity pairs using bivariate probit regression (Stata version 14.2; StataCorp). Bivariate probit regression involves two binary dependent variables (e.g., diarrhea and ALRI) and assesses the likelihood that the two dependent variables occur simultaneously, controlling for site as a fixed effect and clustering within children (as a random effect).^25^ Each bivariate probit model was run, with pairs of symptoms as the outcome variables, after which we compared the correlation between the model residuals. A statistically significant (*P* < 0.05) correlation coefficient suggests that the two symptoms were found together more frequently than would be expected by chance alone.

A Poisson regression model (Proc GLIMMIX, SAS 9.3, SAS Institute, Cary, NC) was used to estimate the risk of incident ALRI and diarrhea as a function of having had diarrhea or fever in the past 30 days (and in the case of the diarrhea outcome, history of ALRI, as well), adjusting for other risk factors and accounting for repeated measures within the same child with a child-level random intercept. The outcomes of interest for the Poisson models were daily incidence of diarrhea or ALRI because of their importance in the global burden of morbidity and mortality as well as their reported interrelationship.^2,4,5^ The model controlled for changing age patterns of illness with the inclusion of a linear spline with seven evenly spaced knots. Seasonality was considered for inclusion but was not found to be necessary in the model. The model controlled for WAZ at the most recent visit as an indicator of recent nutritional status. The mean WAMI (multiplied by 10 for model inclusion) was included to account for household-level factors that relate to environmental enteropathogen exposures^26^; similarly whether a child was first-born was included to address within-household transmission between siblings.^27^ Maternal age (in 5-year increments) was included to control for maternal experience in recognizing and reporting symptoms.^26^ Maternal education was not independently included given that it is a component of the WAMI construct. Child’s sex was included based on sex differences in the risk of illness that have been reported in the literature.^28–32^ The sites were included as fixed effects to adjust for local disease prevalence, and history of hospitalizations was included to adjust for access to health care, as well as to ascertain any short-term morbidity risks associated with recent hospitalization. Whether the child was mostly exclusively breastfed (> 50% of the past month during the first 3 months of life) was also included because of the long-standing evidence that exclusive breastfeeding is associated with lower rates of illness.^13,14,33^ Reported diarrhea, ALRI, and fever in the 30 days before each day of follow-up were included as independent variables in the models. Illness risk factors were evaluated separately for the 0–2, 3–5, and ≥ 6 month age periods. An interaction between morbidities and breastfeeding in the first 3 months of life was included because of observed improvement in Quasi-likelihood under the independence model criterion. The figures in this article were produced using R 3.3.2 (Foundation for Statistical Computing, Vienna, Austria).

## RESULTS

### Cohort description.

More than 80% of the 2,145 children who were enrolled in the cohort were followed for 2 years (range 66–95% among sites, Table 1). Nineteen children from the cohort died during the follow-up, and the suspected causes of death included neonatal disorders, respiratory infections, and diarrhea. Five percent to 33% of the enrolled cohorts dropped out of the study for a variety of reasons (the most common was the family moving out of the study area), with the lowest dropout rates in NEB and the highest in PEL and BRF. Of the 1,731 children included in the analysis, approximately one-third were first-born, except in TZH, where only 9% were first-born children and mothers were, on average, the oldest among the sites (29 compared with the average across all sites of 26 years of age). Baseline WAZ ranged from −4.8 to 2.5, and the mean was lowest in PKN (−1.4). Mean WAMI was lowest in TZH (0.22) and highest in BRF (0.83). On average, children were exclusively breastfed for less than 2 months (range 0.4 months in PKN to 3.4 months in BGD) using the traditional metric of ending exclusive breastfeeding once the child is fed anything other than breast milk.

**Table 1:** Subjects included in the final analysis, *N* (%), and cohort characteristics

		Southern Asia			Latin America		Sub-Saharan Africa		
	BGD	PKN	INV	NEB	BRF	PEL	SAV	TZH	Total
Enrolled	265	277	251	240	233	303	314	262	2,145
Died	3 (1)	8 (3)	2 (1)	0 (0)	0 (0)	2 (0.7)	1 (0.3)	3 (1)	19 (1)
Dropped out	49 (18)	17 (6)	21 (8)	12 (5)	65 (28)	101 (33)	80 (25)	46 (18)	391 (18)
Followed 2 years	213 (80)	252 (91)	226* (90)	228 (95)	168 (72)	199* (66)	232* (74)	213 (81)	1,731* (81)
Cohort characteristics									
Boys (%)	49	51	54	46	46	46	49	49	49
First born (%)	39	22	34	43	31	36	35	9	31
Mean maternal age at baseline (SD)	25 (5)	28 (6)	24 (4)	27 (4)	25 (6)	25 (6)	27 (7)	29 (7)	26 (6)
Mean baseline† WAZ (SD)	−1.27 (0.9)	−1.41 (1.1)	−1.30 (1.0)	−0.91 (1.0)	−0.16 (1.0)	−0.65 (0.9)	−0.36 (0.9)	−0.13 (0.9)	−0.80 (1.1)
Mean WAMI (SD)‡	0.54 (1.2)	0.49 (1.8)	0.48 (1.5)	0.70 (1.3)	0.83 (0.9)	0.54 (1.1)	0.78 (1.0)	0.22 (1.1)	0.57 (2.2)
Mean months of exclusive breastfeeding	3.4	0.4	2.5	1.9	2.3	1.3	1.0	1.5	1.8
Exclusively breastfed > 3 months (%)	58	0.4	39	27	30	17	2	10	22

BGD = Bangladesh—Dhaka; BRF = Brazil—Fortaleza; INV = India—Vellore; NEB = Nepal—Bhaktapur; PEL = Peru—Loreto; SAV = South Africa—Venda; SD = standard deviation; TZH = Tanzania—Haydom; WAMI = water, assets, maternal education, and income; WAZ = weight-for-age *z*-scores.

* Two children did not have any WAMI information (one from Peru and one from South Africa) and two children did not have any information related to their birth order or maternal age (from India) and were, therefore, dropped.

† Children were enrolled and measured within 17 days of birth.

‡ WAMI was assessed up to three times during follow-up and the mean score over the 2-year period was calculated.

### Symptom prevalence.

Among those children followed for 2 years, PKN had the highest mean prevalence of illness reported by the caregiver (72% of days followed) and the highest prevalence for most of the individual symptoms (Table 2). Coughing was the most frequently reported symptom overall, with mean prevalence ranging from 3% in BRF, SAV, and TZH to 27% in PKN. The prevalence of ear pain was, on average, over seven times more common in PKN than in the next highest site (INV). Similarly, mean ALRI prevalence was almost six times more common in PKN than in the next highest site (INV). In five of the study sites, > 90% of the children experienced at least one episode of diarrhea during the first 2 years of life (two sites reported 100%). By contrast, in BRF and SAV only slightly more than half of the children experienced any diarrhea during the first 2 years of life. ALRI was less common than diarrhea in most sites; < 50% of children experienced any ALRI in four sites, and only one site (PKN) had > 90% of children identified as having had an ALRI.

**Table 2:** Mean symptom prevalence (%) in children 0–24 months in the MAL-ED cohort study

Site	BGD	PKN	INV	NEB	BRF	PEL	SAV	TZH
Any symptom	43	47	18	15	4	28	4	7
Diarrhea*	4	9	2	3	1	5	0.4	1
% Children with any diarrhea	97	100	92	94	56	100	59	83
ALRI†	0.3	13	2	1	0.1	0.3	0.3	0.1
% Children with any ALRI	47	99	81	68	18	55	35	45
Vomiting	8	11	2	1	0.2	1	0.4	1
Fever	6	13	6	5	1	4	1	2
Cough	26	27	10	9	3	22	3	3
Ear pain	1	11	1	1	0.1	0.1	0.4	1
Dehydration	0.1	2	0.1	0.1	0	0.3	0	0.1
Decreased appetite	18	0.4	3	0.2	0.1	4	1	3
Decreased activity	0.2	0	1	0.2	0	1	1	2
Caregiver report of illness	53	72	42	16	5	6	5	10
% Symptom days with caregiver report of illness	84	99	96	97	92	19	83	92
% Days with caregiver report of illness with any symptom	67	65	41	88	64	94	79	63

ALRI = acute lower respiratory infections; BGD = Bangladesh—Dhaka; BRF = Brazil—Fortaleza; INV = India—Vellore; NEB = Nepal—Bhaktapur; PEL = Peru—Loreto; SAV = South Africa—Venda; TZH = Tanzania—Haydom.

* The study definition of diarrhea is three or more loose stools in 24 hours or at least one loose stool with blood.

† The study definition of ALRI is met when 1) Child had cough or shortness of breath (today or yesterday) and 2) Rapid respiration rate today (average of two measurements) as defined by a) ≥ 60 breaths/minute when child is < 60 days old, b) ≥ 50 breaths/minute when child is ≥ 60 to < 365 days of age, and c) ≥ 40 breaths/minute when child is ≥ 365 days of age. ALRI episodes are separated by 14 ALRI-“free” days.

### Distribution of symptoms.

**Figure 1.**
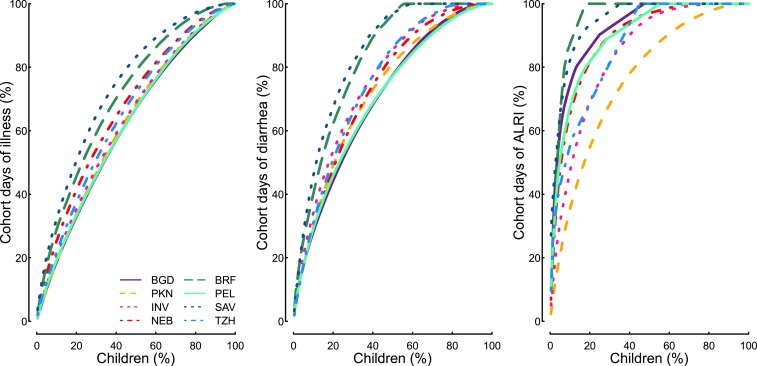
Cumulative distribution function showing percent of children on *x* axis and percent of cohort days with illness (ALRI, diarrhea, cough, fever, or vomiting), diarrhea, and ALRI, by site. ALRI = acute lower respiratory infections; BGD = Bangladesh—Dhaka; BRF = Brazil—Fortaleza; INV = India—Vellore; NEB = Nepal—Bhaktapur; PEL = Peru—Loreto; SAV = South Africa—Venda; TZH = Tanzania—Haydom.

Illnesses were not evenly distributed throughout the population (Figure 1). At the different study sites, between 46% and 65% of children were reported to have 80% of days with illness (combining caregiver reports of ALRI, diarrhea, cough, fever, or vomiting). For diarrhea, between 29% and 54% of children were responsible for 80% of diarrhea days in the cohorts, and for ALRI, between 8% and 43% of children were responsible for 80% of the ALRI days.

Most of the days with defined symptoms coincided with caregiver report of nonspecified “illness,” with the exception of PEL, where only 19% of days with reported symptoms coincided with illness defined by the caregiver (Table 2). On those days when symptoms were reported but the caregiver did not report that the child was ill in PEL, cough was the most common symptom (noted on 83% of those days), followed by diarrhea (noted on 14% of those days). Across the sites, approximately one-third of the caregivers’ reports of child illness did not identify a specific symptom. Most of the symptoms experienced by the children were experienced in isolation (62%) or along with one other symptom (24%) (Supplemental Figure 1). Among the reports of a single symptom, most of the children had cough, followed by fever and diarrhea. Because of the high frequency of cough in the cohort, it was often reported as part of the most common symptom combinations. When adjusting for symptom prevalence using bivariate probit analysis, cough and fever (mean correlation coefficient 0.52), vomiting and fever (0.39), and diarrhea and vomiting (0.39) were the top two-symptom combinations that appeared more often than would be expected by chance (*P* < 0.05) (Supplemental Table 1).

**Figure 2.**
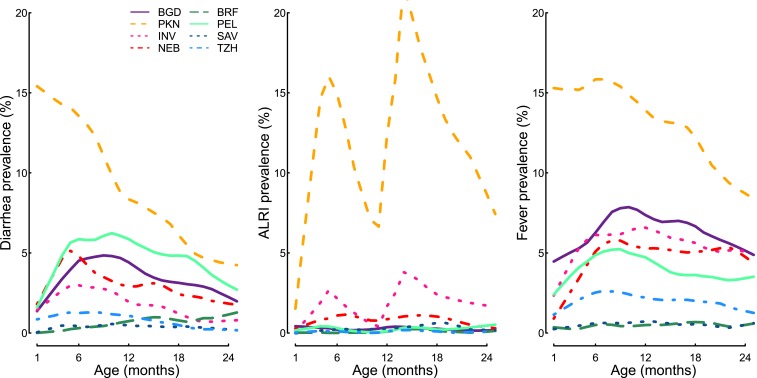
Diarrhea, ALRI, and fever prevalence (%) by age in months and by site. ALRI = acute lower respiratory infections; BGD = Bangladesh—Dhaka; BRF = Brazil—Fortaleza; INV = India—Vellore; NEB = Nepal—Bhaktapur; PEL = Peru—Loreto; SAV = South Africa—Venda; TZH = Tanzania—Haydom.

Monthly diarrhea, ALRI, and fever prevalence were highest in PKN (Figure 2, Supplemental Figure 2). Diarrhea prevalence increased over the first 6 months of life in four of the sites. In the remaining four sites, the mean diarrhea monthly prevalence either stayed low and relatively constant (BRF, SAV, and TZH) or decreased over time (PKN). Two peaks of ALRI monthly prevalence were observed most clearly in PKN and INV (5 and 13 months); these peaks are likely due to the changing definition of ALRI at 12 months of age. The definitional change at 12 months decreases the number of breaths per minute required for ALRI diagnosis from ≥ 50 to ≥ 40.

### Risk of incident diarrhea model.

**Figure 3.**
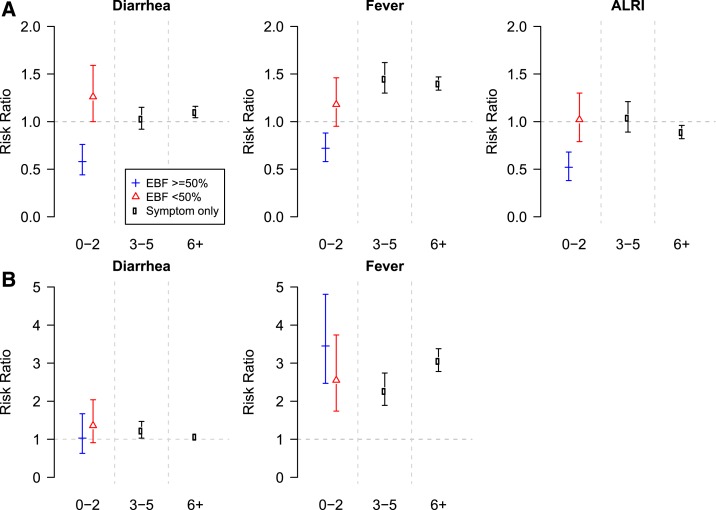
Risk ratio of diarrhea (**A**, figures in top row) and acute lower respiratory infections (ALRI) (**B**, figures in bottom row) associated with history of diarrhea and fever (and ALRI in the diarrhea model) in the past 30 days. Interaction with exclusive breastfeeding (exclusively breastfed for the majority of the past 30 days) was included in the first 3 months of life. Also included in the model are hospitalizations, weight-for-age z-score, water, assets, maternal education, and income, first-born, maternal age, sex, age, and study site, and random effects for child.

Exclusive breastfeeding was protective against incident diarrhea in the first 6 months of life. Children without recent symptoms of diarrhea, ALRI, or fever who were exclusively breastfed for more than half of the past month during the 0–2 and 3–5 month time periods had lower relative risk (RR) of incident diarrhea than did children who were not exclusively breastfed for at least half of the past month (Figure 3; Table 3). When children < 3 months of age experienced illness symptoms (diarrhea, fever, or ALRI in the past 30 days), exclusive breastfeeding was protective against incident diarrhea when compared with children who were not exclusively breastfed in these cohorts.

**Table 3:** Incident diarrhea and ALRI model output

Variable		Diarrhea				ALRI			
		Unadjusted		Adjusted		Unadjusted		Adjusted	
		Relative risk (RR)	95% CI	RR	95% CI	RR	95% CI	RR	95% CI
Hospitalization		1.35*	1.16, 1.57	1.23*	1.06, 1.43	2.48*	2.01, 3.05	1.95*	1.58, 2.41
Weight-for-age z-score		1.02	1.00, 1.05	1.06*	1.03, 1.09	0.99	0.95, 1.03	1.03	0.99, 1.07
Water, assets, maternal education, and income		0.97	0.95, 1.00	0.97*	0.94, 0.99	0.93*	0.90, 0.97	0.94*	0.91, 0.97
First-born		0.95	0.87, 1.03	0.90*	0.83, 0.99	0.89	0.80, 1.00	0.86*	0.76, 0.96
Maternal age (5 year increments)		0.97	0.94, 1.00	0.95*	0.91, 0.98	0.96	0.92, 1.01	0.92*	0.88, 0.97
Sex (girl = 1)		0.92*	0.85, 0.99	0.93	0.86, 0.99	0.83*	0.76, 0.91	0.87*	0.80, 0.96
0–2 months—exclusively breastfed	No symptoms	0.52*	0.45, 0.60	0.39*	0.32, 0.49	1.17	0.92, 1.47	1.11	0.75, 1.66
	Diarrhea	0.86	0.71, 1.05	0.58*	0.44, 0.76	1.56*	1.10, 2.19	1.03	0.63, 1.67
	Fever	0.87	0.72, 1.04	0.72*	0.58, 0.88	3.43*	2.51, 4.69	3.45*	2.47, 4.81
	ALRI	0.93	0.67, 1.30	0.52*	0.35, 0.77	–	–	–	–
0–2 months—not exclusively breastfed	No symptoms	–	–	–	–	–	–	–	–
	Diarrhea	1.26*	1.03, 1.56	1.26*	1.00, 1.59	1.60*	1.11, 2.29	1.36	0.91, 2.04
	Fever	1.32*	1.09, 1.60	1.18	0.95, 1.46	2.94*	2.10, 4.11	2.55*	1.74, 3.74
	ALRI	1.16	0.92, 1.46	1.02	0.80, 1.31	–	–	–	–
3–5 months	Exclusive breastfeeding	0.82*	0.74, 0.91	0.83*	0.75, 0.93	0.78*	0.65, 0.93	0.81*	0.68, 0.98
	Diarrhea	1.09	0.98, 1.21	1.03	0.92, 1.15	1.25*	1.06, 1.47	1.23*	1.03, 1.47
	Fever	1.43*	1.30, 1.58	1.45*	1.30, 1.62	2.37*	1.99, 2.83	2.27*	1.89, 2.74
	ALRI	1.12	1.00, 1.25	1.04	0.92, 1.18	–	–	–	–
6–24 months	Diarrhea	1.13*	1.06, 1.19	1.10*	1.04, 1.16	1.22*	1.12, 1.34	1.07	0.98, 1.17
	Fever	1.38*	1.31, 1.46	1.40*	1.33, 1.47	3.12*	2.84, 3.43	3.06*	2.78, 3.38
	ALRI	0.95	0.89, 1.01	0.89*	0.83, 0.95	–	–	–	–

ALRI = acute lower respiratory infections; CI = confidence interval. Poisson regression models were run for incident diarrhea and ALRI using the same covariates, as well as random effects for child. In addition to the variables shown below, fixed effects were included for age (seven evenly spaced knots) and study site. Unadjusted values only include that variable, age spline, and study site, whereas the adjusted values include all of the other variables in the model.

* *P* < 0.05.

Other factors associated with incident diarrhea include illness, recent hospitalization, and SES. Fever in the previous 30 days was associated with greater RR of incident diarrhea in the 3–5 month period (RR 1.45, 95% confidence interval [CI] 1.30, 1.62). For children older than 6 months of age, diarrhea and fever in the previous 30 days were both associated with higher risk of incident diarrhea (diarrhea: RR 1.10, 95% CI 1.04, 1.16; fever: RR 1.40, 95% CI 1.33, 1.47). Hospitalization in the previous 30 days was associated with higher risk of incident diarrhea (RR 1.23, 95% CI 1.06, 1.43), as was higher WAZ (RR 1.06, 95% CI 1.03, 1.09). Factors that were found to protect against incident diarrhea were higher SES (RR 0.97 per 10% increase in WAMI, 95% CI 0.94, 0.99) and being the child of an older mother (RR 0.95 per 5 years of maternal age, 95% CI 0.91, 0.98).

### Risk of incident ALRI model.

Exclusive breastfeeding was protective against incident ALRI in the 3- to 5-month period (RR 0.81, 95% CI 0.68, 0.98), but had no apparent relationship with ALRI in the first 3 months of life (RR 1.11, 95% CI 0.75, 1.66) (Figure 3). In addition, no relationship was observed between exclusive breastfeeding and history of symptoms on the risk of ALRI in the first 3 months of life.

Fever and recent hospitalization were two of the strongest risk factors for incident ALRI in the cohort. Fever in the previous month was consistently associated with increased chance of ALRI in all age groups (0–2 EBF RR 3.45, 95% CI 2.47, 4.81; 0–2 not EBF: RR 2.55, 95% CI 1.74, 3.74; 3–5: RR 2.27, 95% CI 1.89, 2.74; > 6: RR 3.06, 95% CI 2.78, 3.38). Diarrhea was associated with a higher risk of ALRI only in the 3- to 5-month age group (RR 1.23, 95% CI 1.03, 1.47). Hospitalization in the past month was associated with a 1.95 times higher risk of incident ALRI (95% CI 1.58, 2.41), and girls had a lower incidence of ALRI than boys (RR 0.87, 95% CI 0.80, 0.96). Higher WAMI was protective of incident ALRI (RR 0.94, 95% CI 0.91, 0.97), as was older maternal age (RR 0.92, 95% CI 0.88, 0.97). Children who did not have older siblings were also less likely to have incident ALRI (RR 0.86, 95% CI 0.76, 0.96). ALRI in the previous month appeared to be protective against incident ALRI, likely for definitional reasons (the requirement of 14 ALRI-free days to define a new episode reduces by almost half the number of days in which ALRI can be diagnosed in any 30-day period), and therefore, was not included in the final model.

## DISCUSSION

The MAL-ED study is a comprehensive cohort study that collected detailed data on a variety of symptoms across the first 2 years of life in eight disparate sites. Symptoms were not evenly distributed among the children in the eight study populations. The majority of illness symptoms (80%) were concentrated in around half of the children and although the distribution of diarrhea followed that of all grouped illnesses, ALRI were more highly concentrated in as few as 8% of children. Both BRF and SAV had the most skewed concentration of symptoms in the fewest children, but equally had the fewest reported days of illness. This is unlikely to reflect any disparity between maternal and trained fieldworker reports, which were highly correlated in most sites. Most of the symptoms existed on their own or along with one other symptom; three or more concurrent symptoms were seen on 14% of the days with any symptoms, with the highest percentages in PKN and TZH (Supplemental Figure 1).

Exclusive breastfeeding in the first 6 months of life was protective against incident diarrhea, and in the 3- to 5-month period was protective against incident ALRI in the MAL-ED cohort. Children who were exclusively breastfed for at least half of the previous 30 days were less likely to experience diarrhea, and this was true whether they had experienced other illnesses in the past 30 days. The potential mechanisms by which exclusive breastfeeding may protect against diarrhea and ALRI include the immune-boosting properties of breastfeeding itself and, for diarrhea, reduced exposure to contaminated water or food.^33,34^ The findings reinforce the importance of exclusive breastfeeding as a method to prevent diarrhea and ALRI in early childhood.

Previous studies have reported on the importance of breastfeeding for reducing morbidity and mortality due to pneumonia^9,12,14^ and diarrhea.^7–13^ In this cohort, < 60% of children were exclusively breastfed beyond 1 month of age when strictly defining the end of exclusive breastfeeding at the first time other foods or drinks are introduced.^22^ However, it has been noted in this cohort that mothers may introduce non-breast milk items to the diet and then return to exclusive breastfeeding.^23^ Considering a more standard approach to exclusive breastfeeding (strictly defining exclusively breastfed children as not having received any non-breast milk item in their diet) resulted in most children falling into the non-exclusively breastfed group. For that reason, we chose to compare the risk of illness in children who were exclusively breastfed more than half of the days in the past month with that of children who were given non-breast milk food or liquids more than half of the days in the past month. Even with this more relaxed definition of exclusive breastfeeding, we identified protective effects of exclusive breastfeeding on incidence of diarrhea and ALRI.

This study has demonstrated that recent history of diarrhea is a risk factor for incident diarrhea, a finding which is supported by similar observations in the literature.^28,35–37^ Diarrhea in the past 30 days was a risk factor for incident diarrhea in the 6- to 24-month age group, as well as in the first 3 months of life among children who were not exclusively breastfed the majority of the past month. It is possible that children who have recurrent diarrhea do so because of child-specific factors, such as gut dysfunction resulting from previous enteropathogen exposure,^38^ decreased immune function due to nutrient deficiency (e.g., zinc),^39^ or noninfectious factors, such as allergies or irritable bowel syndrome.^40^ In addition, there might be household factors related to water and sanitation or other means of exposure to pathogens that might result in recurrent diarrhea in a child. More targeted experimental research would be needed to elucidate those factors that result in recurrent diarrhea to decrease the childhood morbidity and mortality burden in these communities.

Diarrhea has been found to be a risk factor for incident ALRI in previous studies,^4–6,41^ as well as in this study, although the association was statistically significant only in the 3- to 5-month age group. Short-term effects of diarrhea, including zinc losses and immune dysfunction, as well as the more general or long-term potential effects of diarrhea, such as stunting, are some of the pathways posited to explain this relationship. Unlike the other works published on this relationship, we included both diarrhea and fever in the same model, and when fever was controlled for in the analysis, the diarrhea relationship became nonsignificant (Table 3). Two of the previous studies that found these relationships considered the number of days with diarrhea in the previous period,^4,5^ whereas we considered diarrhea history to be a binary exposure variable (yes or no in the previous month) due to sample size constraints. This categorization of diarrhea history did not allow us to observe the effects that others have described for each additional day of diarrheal disease.

With respect to other risk factors concerned, fever was a consistent and strong risk factor for both incident diarrhea and ALRI. It is likely that caregivers observed health changes before the onset of diarrhea or ALRI symptoms, with fever being one of the clearer symptoms that caregivers would recall when describing illness histories. Hospitalization within the past 30 days was also associated with greater risk of both diarrhea and ALRI in all age groups. A child who has been hospitalized may be more likely to be exposed to enteric or respiratory pathogens while hospitalized and more susceptible (because they have been severely ill). In addition, children who have had a recent illness or were hospitalized may have received antibiotics that can cause antibiotic-associated diarrhea. Higher WAZ was also associated with a small but significantly higher risk of diarrhea. This counter-intuitive finding deserves further study but we hypothesize that weight loss is associated with having diarrhea; therefore, one might be heavier before a diarrhea episode than during or immediately after an episode.

Some protective factors were identified in this analysis. Higher SES (WAMI) and older maternal age were protective against both diarrhea and ALRI. Households with higher SES have better access to water and sanitation, as well as presumably better access to good nutrition and other health-promoting factors, thereby decreasing risk of diarrhea and ALRI. Older mothers are also likely to have greater access to resources, which may reduce exposure to infectious diseases. First-born children were less likely to experience diarrhea and ALRI, although the relationship was only statistically significant for ALRI. It is likely that older siblings bring infections into the household, for example, respiratory infections. Consistent with previous findings,^31,32^ girls were less likely to experience ALRI than boys, attributed to boys’ smaller airway size at the same ages, adjusted for size.^42^

These findings confirm previous reports that children who have had an episode of diarrhea are at greater risk of experiencing additional episodes and, given that they then contribute more to estimates of disease prevalence and potentially spread more infectious agents into the environment, should be targeted for public health programs. In addition, diarrhea is associated with a higher risk of ALRI; therefore, diarrhea-prevention programs (e.g., water and sanitation) can potentially have an impact not only on diarrhea burden in a community, but also can reduce the burden of ALRI. Finally, exclusive breastfeeding for the first 6 months of life has the potential to protect against both diarrhea and ALRI in early childhood, while providing complete nutrition to the child. Continued and strengthened promotion of exclusive breastfeeding during the first 6 months of life is recommended to decrease morbidity and mortality, with the ultimate goal of promoting adequate growth and development.

## APPENDIX

### MAL-ED investigators.

Angel Mendez Acosta,^1^ Rosa Rios de Burga,^1^ Cesar Banda Chavez,^1^ Julian Torres Flores,^1^ Maribel Paredes Olotegui,^1^ Silvia Rengifo Pinedo,^1^ Mery Siguas Salas,^1^ Dixner Rengifo Trigoso,^1^ Angel Orbe Vasquez,^1^ Imran Ahmed,^2^ Didar Alam,^2^ Asad Ali,^2^ Zulfiqar A. Bhutta,^2^ Shahida Qureshi,^2^ Muneera Rasheed,^2^ Sajid Soofi,^2^ Ali Turab,^2^ Anita K. M. Zaidi,^2^ Ladaporn Bodhidatta,^3^ Carl J. Mason,^3^ Sudhir Babji,^4^ Anuradha Bose,^4^ Ajila T. George,^4^ Dinesh Hariraju,^4^ M. Steffi Jennifer,^4^ Sushil John,^4^ Shiny Kaki,^4^ Gagandeep Kang,^4^ Priyadarshani Karunakaran,^4^ Beena Koshy,^4^ Robin P. Lazarus,^4^ Jayaprakash Muliyil,^4^ Mohan Venkata Raghava,^4^ Sophy Raju,^4^ Anup Ramachandran,^4^ Rakhi Ramadas,^4^ Karthikeyan Ramanujam,^4^ Reeba Roshan,^4^ Srujan L. Sharma,^4^ Shanmuga Sundaram E.,^4^ Rahul J. Thomas,^4^ William K. Pan,^5,6^ Ramya Ambikapathi,^6^ J. Daniel Carreon,^6^ Vivek Charu,^6^ Viyada Doan,^6^ Jhanelle Graham,^6^ Christel Hoest,^6^ Stacey Knobler,^6^ Dennis R. Lang,^6,7^ Benjamin J. J. McCormick,^6^ Monica McGrath,^6^ Mark A. Miller,^6^ Archana Mohale,^6^ Gaurvika Nayyar,^6^ Stephanie Psaki,^6^ Zeba Rasmussen,^6^ Stephanie A. Richard,^6^ Jessica C. Seidman,^6^ Vivian Wang,^6^ Rebecca Blank,^7^ Michael Gottlieb,^7^ Karen H. Tountas,^7^ Caroline Amour,^8^ Eliwaza Bayyo,^8^ Estomih R. Mduma,^8^ Regisiana Mvungi,^8^ Rosemary Nshama,^8^ John Pascal,^8^ Buliga Mujaga Swema,^8^ Ladislaus Yarrot,^8^ Tahmeed Ahmed,^9^ A. M. Shamsir Ahmed,^9^ Rashidul Haque,^9^ Iqbal Hossain,^9^ Munirul Islam,^9^ Mustafa Mahfuz,^9^ Dinesh Mondal,^9^ Fahmida Tofail,^9^ Ram Krishna Chandyo,^10^ Prakash Sunder Shrestha,^10^ Rita Shrestha,^10^ Manjeswori Ulak,^10^ Aubrey Bauck,^11^ Robert E. Black,^11^ Laura E. Caulfield,^11^ William Checkley,^6,11^ Margaret N. Kosek,^11^ Gwenyth O. Lee,^11^ Kerry Schulze,^11^ Pablo Peñataro Yori,^11^ Laura E. Murray-Kolb,^12^ A. Catharine Ross,^12^ Barbara Schaefer,^6,12^ Suzanne Simons,^12^ Laura Pendergast,^13^ Cláudia B. Abreu,^14^ Hilda Costa,^14^ Alessandra Di Moura,^14^ José Quirino Filho,^6,14^ Alexandre Havt,^14^ Álvaro M. Leite^14^, Aldo A. M. Lima,^14^ Noélia L. Lima,^14^ Ila F. Lima,^14^ Bruna L. L. Maciel,^14^ Pedro H. Q. S. Medeiros,^14^ Milena Moraes,^14^ Francisco S. Mota,^14^ Reinaldo B. Oriá,^14^ Josiane Quetz,^14^ Alberto M. Soares,^14^ Rosa M. S. Mota,^14^ Crystal L. Patil,^16^ Pascal Bessong,^17^ Cloupas Mahopo,^17^ Angelina Maphula,^17^ Emanuel Nyathi,^17^ Amidou Samie,^17^ Leah Barrett,^18^ Rebecca Dillingham,^18^ Jean Gratz,^18^ Richard L. Guerrant,^18^ Eric Houpt,^18^ William A. Petri Jr.,^18^ James Platts-Mills,^18^ Rebecca Scharf,^18^ Binob Shrestha,^19^ Sanjaya Kumar Shrestha,^19^ Tor Strand,^15,19^ and Erling Svensen.^8,20^

### Institutions.

^1^A.B. PRISMA, Iquitos, Peru; ^2^Aga Khan University, Karachi, Pakistan; ^3^Armed Forces Research Institute of Medical Sciences, Bangkok, Thailand; ^4^Christian Medical College, Vellore, India; ^5^Duke University, Durham, NC; ^6^Fogarty International Center/National Institutes of Health, Bethesda, MD; ^7^Foundation for the NIH, Bethesda, MD; ^8^Haydom Lutheran Hospital, Haydom, Tanzania; ^9^icddr,b, Dhaka, Bangladesh; ^10^Institute of Medicine, Tribhuvan University, Kathmandu, Nepal; ^11^Johns Hopkins University, Baltimore, MD; ^12^The Pennsylvania State University, University Park, PA; ^13^Temple University, Philadelphia, PA; ^14^Universidade Federal do Ceara, Fortaleza, Brazil; ^15^University of Bergen, Bergen, Norway; ^16^University of Illinois at Chicago, Chicago, IL; ^17^University of Venda, Thohoyandou, South Africa; ^18^University of Virginia, Charlottesville, VA; ^19^Walter Reed/AFRIMS Research Unit, Kathmandu, Nepal; ^20^Haukeland University Hospital, Bergen, Norway.

## Supplementary Material

Supplemental Figures and Table.
