# Supplementary material for: Relationships among Common Illness Symptoms and the Protective Effect of Breastfeeding in Early Childhood in MAL-ED: An Eight-Country Cohort Study
Source: Am J Trop Med Hyg. 2018 Jan 29;98(3):904–12. doi: 10.4269/ajtmh.17-0457 (PMC5930868; doi:10.4269/ajtmh.17-0457)
Supplement: Supplementary file 1 [file tpmd170457.SD1.pdf]

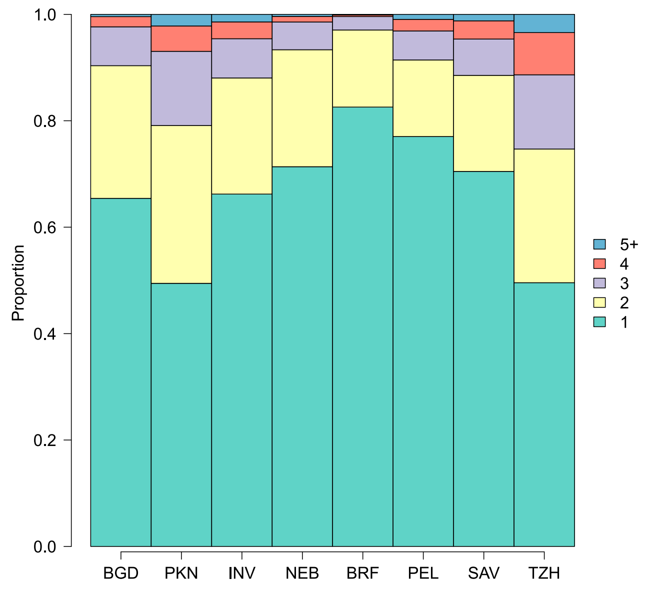

SUPPLEMENTAL FIGURE 1. Proportion of symptom days with 1 to 5+ symptoms identified, by study site.

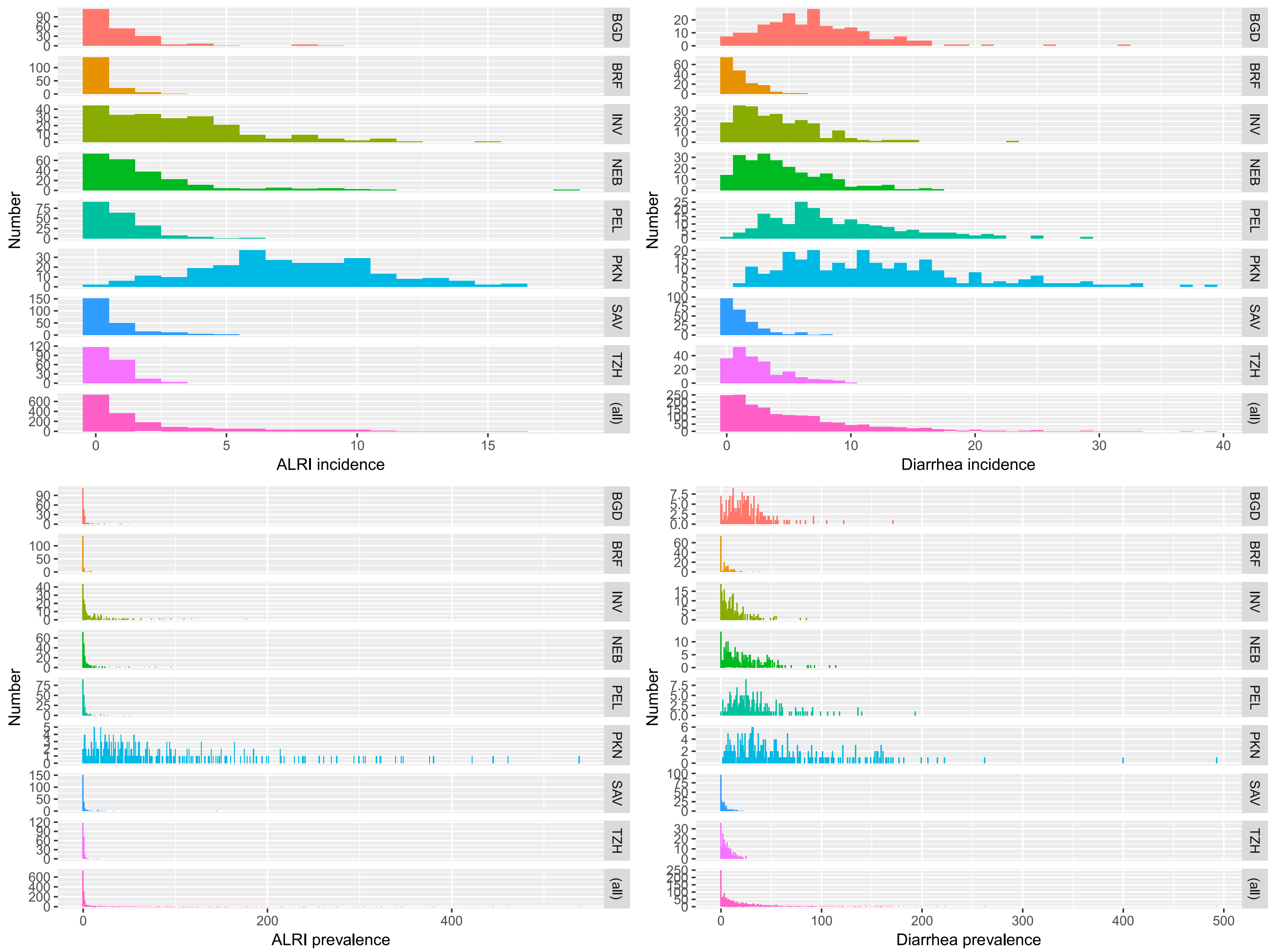

SUPPLEMENTAL FIGURE 2. Incidence and prevalence (per child year) of diarrhea and ALRI in the first 2 years of life in the MAL-ED cohort. ALRI = acute lower respiratory infections; BGD = Bangladesh—Dhaka; BRF = Brazil—Fortaleza; INV = India—Vellore; NEB = Nepal—Bhaktapur; PEL = Peru—Loreto; SAV = South Africa—Venda; TZH = Tanzania—Haydom.

Supplemental Table 1

Correlation coefficients between the residuals of symptoms using bivariate probit analysis with clustering by child (adjusting for site)

First symptom	Second symptom	Rho adjusting for site*	*N*
Cough	Fever	0.52	32,682
Vomiting	Fever	0.39	10,479
Diarrhea	Vomiting	0.39	7,350
ALRI	Fever	0.30	6,933
Diarrhea	Fever	0.30	8,099
ALRI	Vomiting	0.24	4,897
Diarrhea	Cough	0.18	12,725
Diarrhea	ALRI	0.16	3,533

ALRI = acute lower respiratory infections.
* All P values < 0.0001.
